# Associations Between Sputum Torque Teno Virus Load and Lung Function and Disease Severity in Patients With Chronic Obstructive Pulmonary Disease

**DOI:** 10.3389/fmed.2021.618757

**Published:** 2021-04-22

**Authors:** Yu Xie, Qing Xue, Weike Jiao, Jianhui Wu, Yan Yu, Lili Zhao, Yu Xu, Xinyu Deng, Guiju Fang, Yali Zheng, Zhancheng Gao

**Affiliations:** ^1^Department of Respiratory and Critical Care Medicine, Peking University People's Hospital, Beijing, China; ^2^Department of Pulmonary and Critical Care Medicine, Ningde Municipal Hospital Affiliated to Fujian Medical University, Ningde, China; ^3^Department of Respiratory, Critical Care, and Sleep Medicine, Xiang'an Hospital of Xiamen University, Xiamen, China

**Keywords:** chronic obstructive pulmonary disease, torque teno virus, lung function, disease severity, biomarker

## Abstract

**Purpose:** Viral load of Torque Teno virus (TTV) is elevated in immunosuppressed patients. The weakened immune response is typical in chronic obstructive pulmonary disease (COPD) patients. However, the relationship between TTV and COPD is still unknown.

**Patients and Methods:** We enrolled 91 patients admitted to hospitals with acute exacerbation of COPD (AECOPD) between January 2017 and August 2017 (ClinicalTrials.gov ID, NCT03236480). Sputum samples were gathered during hospitalization and the 120-day follow-up. TTV distribution and genogroups were assessed, and the associations between viral loads and clinical parameters were analyzed.

**Results:** TTV DNA was detected in 95.6% of COPD patients, and the viral load was nearly invariable at the stable and exacerbation states. Most TTV DNA-positive patients carried four distinct genotypes. Sputum load of TTV was positively associated with RV/TLC (*r* = 0.378, *p* = 0.030), and negatively correlated with FEV1/pre and FEV1/FVC (*r* = −0.484, −0.432, *p* = 0.011, 0.024, respectively). Neutral correlation between the TTV DNA load and COPD assessment test (CAT) scores (*r* = 0.258, *p* = 0.018) was observed.

**Conclusion:** Sputum loads of TTV DNA could be a novel indicator for lung function and disease severity assessment in COPD patients.

## Introduction

Torque teno virus (TTV), a DNA virus, was initially isolated from the serum specimen of a patient who developed hepatitis of unknown etiology after blood transfusion (1). TTV belongs to the Anelloviridae family and has considerable genetic variability and extreme diversity (2, 3), and at least five genogroups and 23 genotypes have been identified (4, 5). TTV viremia was first detected in patients with liver diseases and blood donors, renal failure, and others (6–8). Then, increased sensitivity of viral detection reveals high prevalence of TTV viremia in the human population, even in healthy individuals, ranging from 14.2 to 100% (9–11). The exact role of TTV is yet to be established. Studies demonstrate a connection between TTV and host immune status. The TTV level can be used to determine the degree of immunosuppression (12), and high TTV DNA levels reflect severe immunosuppression (13). Among human immunodeficiency virus (HIV)-infected patients, higher TTV DNA titers were associated with lower CD4^+^ cell counts and decreased survival (14). Besides, TTV infection may play a potential role in pulmonary diseases. Research has shown that the original infection site may be the respiratory tract, which may be a site of continual replication (15). TTV can infect the ciliated respiratory cells (16) and may contribute to lung impairment in asthma (17).

Chronic obstructive pulmonary disease (COPD), a chronic airway inflammation, is characterized by a progressive and irreversible decline in lung function, and recurrent episodes of respiratory infection. Suppressed innate and adaptive immune responses to the respiratory pathogen are strongly associated with the acute exacerbation of COPD (AECOPD) and disease pathogenesis (18–20). Hence, we hypothesize that the presence of TTV might be related to the progression of COPD, and the TTV load may indicate the immune status of COPD patients. In the current study, we investigated the distribution and the genotypic characteristics of TTV in COPD patients and explored the relationships between TTV viral load and the clinical parameters of COPD.

## Materials and Methods

### Patients

The study enrolled 91 patients (M = 86, F = 5, mean age of 71 ± 8 years) with AECOPD between January 2017 and August 2017 (ClinicalTrials.gov ID, NCT03236480) who were consecutively hospitalized at Ningde Hospital, Fujian, China and Peking University People's Hospital, Beijing, China. The diagnosis and assessment of the severity of COPD were made according to the recommendations of the Global Initiative for Chronic Obstructive Lung Disease (GOLD) committee (21). Specifically, for patients hospitalized before May 1st, 2017 (cohort A, *n* = 65), clinical data during hospitalization were collected using a standard electronic medical record. For patients enrolled after May 1st, 2017 (cohort B, *n* = 26), additional follow-up for 120 days was requested. Patients completed the follow-up forms (including questions about smoking status, symptoms, medications and exacerbations) every 30 days during follow-up. The detail of treatment before the onset and medication use during hospitalization is shown in Table 1. When patients were hospitalized and clinically stable, spirometry was performed in 87 patients. Meanwhile, at the third follow-up, spirometry was performed again in 30 patients. We collected laboratory and clinical data from medical records by using spreadsheets. The Ethics Committee of Peking University People's Hospital approved the study (Approval number: 2016PHB202-01). This trial was conducted in accordance with the Declaration of Helsinki. Patients all gave written informed consent before their participation in the study.

**Table 1:** Epidemiological and clinical manifestations of patients with different GOLD grades (*n* = 91).

**Characteristics**	**GOLD $\frac{1}{2}$ (*n* = 13)**	**GOLD 3 (*n* = 32)**	**GOLD 4 (*n* = 46)**	***p-*value**
Age^a^	74.1 ± 6.3	73.2 ± 9.9	70.1± 7.9	0.170
Male	12 (92.3%)	29 (90.6%)	45 (97.8%)	0.336
BMI^a^, kg/m^2^	21.2 ± 2.4	20.9 ± 2.7	21.7 ± 3.2	0.567
Pack-years	30 (0–45)	30 (0–40)	30 (23–48)	0.580
Current smoker	7 (53.8%)	14 (43.8%)	12 (26.1%)	0.101
**Comorbidities**				
Diabetes mellitus	1 (7.7%)	4 (12.5%)	7 (15.2%)	0.770
Hyperlipidemia	0 (0.0%)	0 (0.0%)	3 (6.5%)	0.382
Coronary heart disease	5 (38.5%)	6 (18.8%)	13 (28.3%)	0.364
Chronic kidney disease	0 (0.0%)	2 (6.3%)	0 (0.0%)	0.242
Eosinophils / %	3.0 (0.9–3.5)	1.3 (0.4–3.6)	0.7 (0.2–2.2)	0.068
FeNO^b^/ppb				0.190
≤25	6 (46.1%)	16 (53.3%)	25 (62.5%)	
25–50	5 (38.5%)	13 (43.3%)	13 (32.5%)	
≥50	2 (15.4%)	1 (3.3%)	2 (5.0%)	
Bronchodilator/steroid combination preparation use during the stable period	11 (84.6%)	21 (65.6%)	40 (87.0%)	0.065
Inhaled bronchodilators use during the stable period	0 (0.0%)	1 (3.1%)	2 (4.3%)	1.000
Glucocorticoid use during acute exacerbation	8 (61.5%)	28 (87.5%)	45 (97.8%)	0.001
Antibiotic use during acute exacerbation	11 (84.6%)	30 (93.8%)	45 (97.8%)	0.171
Acute exacerbation in previous 12 months	12 (92.3%)	30 (93.8%)	44 (95.7%)	0.838
6 MWD^b^				<0.001
≥350 m	3 (25.0%)	0 (0.0%)	0 (0.0%)	
250–349 m	9 (75.0%)	19 (59.4%)	8 (17.8%)	
150–249 m	0 (0.0%)	7 (21.9%)	23 (51.1%)	
≤149 m	0 (0.0%)	6 (18.8%)	14 (31.1%)	
mMRC^b^				<0.001
1	3 (23.1%)	0 (0.0%)	0 (0.0%)	
2	7 (53.8%)	17 (53.1%)	9 (20.0%)	
3	3 (23.1%)	14 (43.8%)	35 (77.8%)	
4	0 (0.0%)	1 (3.1%)	1 (2.2%)	
CAT^a^	14.15 ± 3.85	18.88 ± 5.33	21.60 ± 3.86	<0.001
**Outcome**				
Type I Respiratory failure	3 (23.1%)	7 (21.9%)	7 (15.2%)	0.690
Type II Respiratory failure	3 (23.1%)	9 (28.1%)	12 (26.1%)	0.939
Noninvasive ventilation	0 (0.0%)	6 (18.8%)	18 (39.1%)	0.009
Tracheal intubation	0 (0.0%)	0 (0.0%)	2 (4.3%)	0.641
In-hospital mortality	0 (0.0%)	0 (0.0%)	2 (4.3%)	0.641
Length of stay, days	13 (9–20)	11 (10–15)	13 (11–16)	0.555

*Clinical values represent the mean ± standard deviation (SD) or median values (interquartile range, IQR). Continuous values were compared by using the Kruskal-Wallis test, and Fisher's exact test or the chi-square test were used to assess proportions between the groups, unless otherwise indicated*.

a *Variables were analyzed by using one-way analysis of variance (ANOVA)*.

b *Variables were analyzed by using The chi-square test for trend*.

*BMI, body mass index; 6 MWD, 6-min walk distance; mMRC, modified British medical research council; CAT, COPD assessment test*.

### Sputum Specimens

Briefly, sputum samples were collected during hospitalization and follow-up, including 24 h after admission, 24 h before discharge and every 30 days during the 120-day follow-up. Sputum was discharged into a sterile cup and then incubated with 1× volume 0.1% dithiothreitol (DTT) solution at 37°C for 30 min. Samples were mixed with an equal volume (compared to the DTT solution) of sterile normal saline, shaken for 5 min, and then centrifuged at 12,000 rpm for 10 min at room temperature. The precipitate was cryopreserved at −80°C until analysis. Sputum DNA was extracted from 200 μl samples using the QIAamp DNA Mini Kit (QIAgen, Valencia, CA) according to the instructions of the manufacturer. The quality control of the extracted DNA was done using the NanoDrop ONEc spectrophotometer (Thermo Fisher Scientific, USA). We detected and quantified the TTV DNA load in samples obtained at admission, discharge, and every 30 days during follow-up.

### TTV Quantification and Genetic Characterization

TaqMan real-time PCR amplification and the standard curve method were used to measure the TTV loads. The quantitative assays were performed using a 7500 Real-Time PCR System under the following conditions: 95.0°C for 30 s, followed by 40 amplification cycles of 5 s at 95.0°Cand 30 s at 60.0°C (Applied Biosystems, Foster City, CA). We designed primers and probes according to the relatively conserved PCR sequence (EcoRI to HindIII, 251 bp) of the TTV genome (pcDNA™ 3.1/myc-His(-) A plasmid) and used them to generate the standard curve. Because this PCR was based on a remarkably conserved segment of the viral noncoding region, its detection encompassed all the genotypes of TTV placed in GenBank. The DNA sequences obtained from PCR were identified as TTV sequence by BLAST (Basic Local Alignment Search Tool; NCBI home page) comparison. The Ct values of the standard curve were obtained using seven 10-fold dilutions. The Ct values generated by the 10-fold dilution sequence were regressed, and the correlation coefficient was 0.99. The quantification limit of the system was 3.65–9.65 copies per ml. Negative controls contained only the PCR reaction solution. Specimens of patients were tested at least in duplicate. Since only two sputum specimens were collected and tested in the fourth follow-up after discharge, they were excluded in the subsequent analysis.

Nested PCR was performed to detect the TTV DNA genogroups for the TTV-DNA-positive samples using the group-specific primers AB017610, AF261761, AB028669, AB038624 and AB064606, as described by previous studies (15, 22). We have examined the DNA sequences obtained from each primer using BLAST (NCBI home page) and were identified as group-specific TTV sequences. The evaluation of specificity and sensitivity of the genogroup assay have been measured elsewhere previously (15, 22). The first and second rounds of amplification used an initial denaturing temperature at 94°C for 3 min, followed by 30 cycles of 30 s at 94°C to denature the double-stranded DNA, 30 s at 63°C (genogroup 1), 55°C (genogroup 2), 65°C (first round) and 55°C (second round) (genogroup 3), 65°C (genogroup 4), or 60°C (genogroup 5) for annealing, and 1 min at 72°C for extension, and a final extension of 5 min at 72°C. We observed the amplified product by electrophoresis with a 0.8% agarose gel at 120 V for 30 min, and we stained the gel with ethidium bromide.

### Statistical Analysis

IBM SPSS Statistics 25.0 (IBM, 2017) and GraphPad Prism version 7.0 were used for statistical analysis. The Shapiro-Wilk test was used to assess the normality of clinical data and TT virus-related data obtained from quantification and genetic characterization. Continuous variables that conform to normal distribution are presented as the mean ± standard deviation (SD), and continuous variables conform to a non-normal distribution are presented as median values and interquartile ranges (IQRs) (25th and 75th percentiles). Categorical variables are presented as numbers (percentages). Student's *t*-test or one-way analysis of variance (ANOVA) were used to analyze continuous data that conform to normal distribution, and Mann-Whitney U or Kruskal-Wallis test were used to analyze continuous data that conform to non-normal distribution. Fisher's exact test, the chi-square test, or the chi-square test for trend were used to analyze categorical data. Spearman's rank test was performed to analyze the correlations between TTV DNA load and clinical parameters. Differences were considered significant at two-sided *p*-value < 0.05.

## Results

We detected TTV DNA in 240 sputum specimens obtained at admission and discharge and during follow-up. Sputum specimens were obtained from 91 patients who were hospitalized because of AECOPD. TTV DNA was positive in the vast majority of patients (95.6%, 87/91).

### Epidemiological and Clinical Characteristics

The patients with AECOPD were grouped according to the recommendations of the Global Initiative for Chronic Obstructive Lung Disease (GOLD) committee. In general, GOLD 1, 2, 3, and 4 grades were defined as FEV1 ≥ 80% predicted, 50% ≤ FEV1 < 80% predicted, 30% ≤ FEV1 < 50% predicted, and FEV1 < 30% predicted, respectively (21). The clinical and biological manifestations of patients based on GOLD grades are shown in Table 1. A significant difference was observed in the disease severity between groups. The higher the GOLD grade, the more severe the disease was. Specifically, there were significantly differences between groups in 6-min walk distance (6 MWD) (*p* < 0.001), COPD assessment test (CAT) score (*p* < 0.001), modified British medical research council (mMRC) score (p < 0.001), and noninvasive ventilation (*p* = 0.009). There was no critically significant association between the GOLD grade and patient age, body mass index (BMI), pack-years, comorbidities, acute exacerbation, and outcome (noninvasive ventilation excluded).

### TTV Is Common in COPD Patients

The prevalence of TTV was 85.1% (74/87) in patients at admission and 96.6% (85/88) in patients at discharge. During the study period, 100% (22/22), 100% (21/21), and 90.9% (20/22) of patients were TTV DNA-positive when screened 1, 2, and 3 months after discharge, respectively. The detailed distribution of TTV detection is shown in Table 2.

**Table 2:** Distribution of TTV detection in patients (*n* = 91).

**Time points**	**N**	**No. Positive (%)**	**TTV Load (log_**10**_ DNA copies/ml)**
Admission	87	74 (85.1%)	3.5 (2.9–4.2)
Discharge	88	85 (96.6%)	3.6 (3.0–4.4)
1 month after discharge	22	22 (100%)	3.5 ± 0.7
2 months after discharge	21	21 (100%)	3.2 ± 0.7
3 months after discharge	22	20 (90.9%)	3.3 ± 1.0

*Clinical values represent the mean ± standard deviation (SD) or median values (interquartile range, IQR)*.

*TTV, Torque Teno virus*.

**Figure 1:**
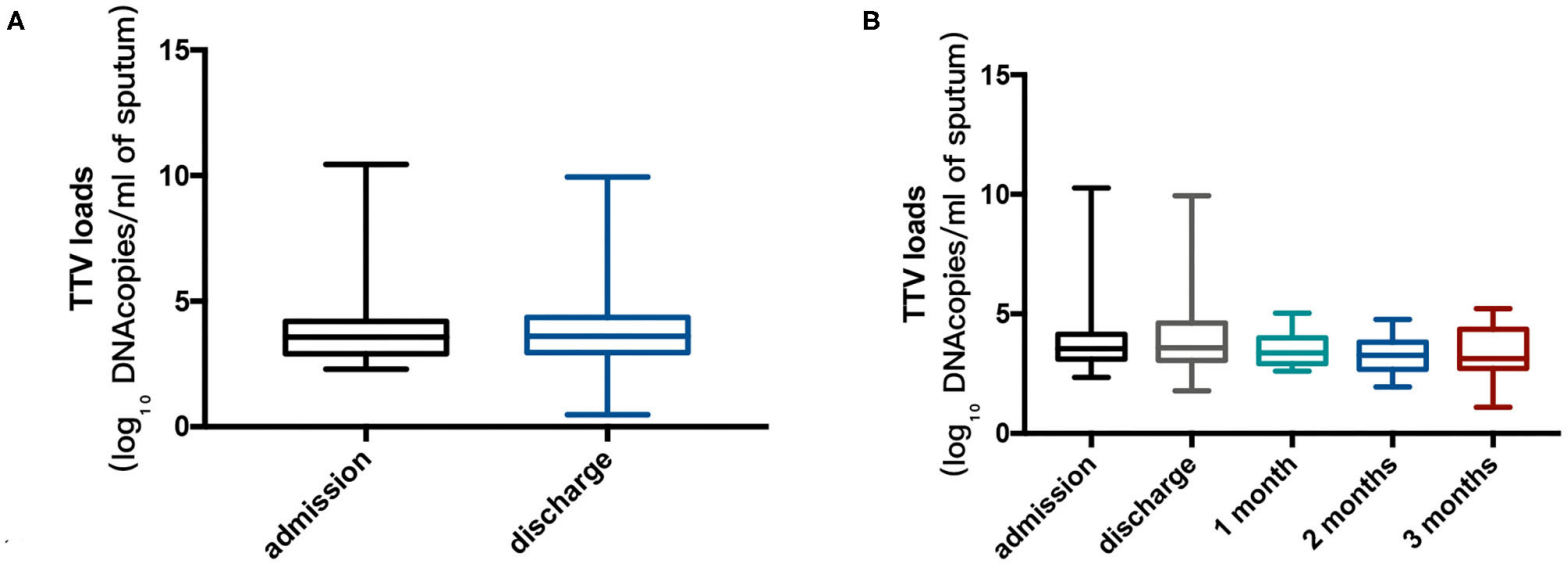
**(A)** Torque Teno virus (TTV) DNA log_10_ sputum loads at admission for 74 patients and at discharge for 85 patients. The Mann-Whitney U test was performed to compare the median values, and no remarkable differences were found between the two points (*p* = 0.870). **(B)** TTV DNA log_10_ sputum loads at admission, at discharge and during follow-up (22, 21, and 20 patients at 1, 2, and 3 months after discharge, respectively) in cohort B. Kruskal-Wallis test was performed to compare the median values, and no significant differences were found between the points in time (*p* = 0.270). Thick horizontal bars represent the median value of each point.

The median TTV DNA load was 3.5 log_10_ copies/ml (IQR 2.9–4.2 log_10_ copies/ml) and 3.6 log_10_ copies/ml (IQR 3.0–4.4 log_10_ copies/ml) at admission and at discharge, respectively. There was a slight decrease in TTV DNA load during follow-up, and the difference did not show statistical significance. The viral loads during the study period are shown in Figure 1.

### Sputum Load of TTV DNA Is Related to Lung Function

**Figure 2:**
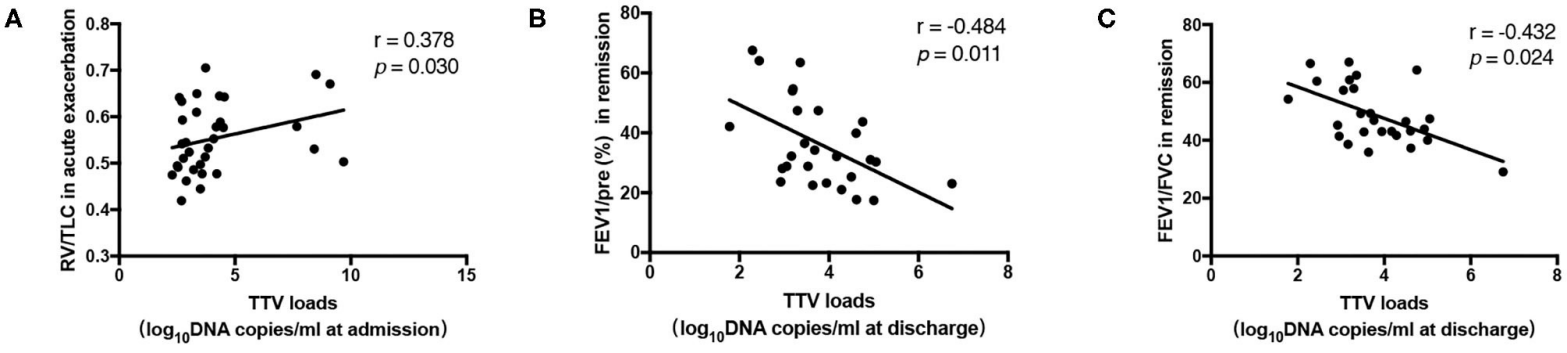
**(A)** Correlation between the Torque Teno virus (TTV) DNA load and the residual volume (RV)/total lung capacity (TLC) in 33 patients. **(B,C)** Correlation between the TTV DNA load and forced expiratory volume in 1 s (FEV1)/pre, FEV1/forced vital capacity (FVC) in 27 patients. The correlations between the values were assessed by the Spearman's rank test. The *r* and *p*-values are shown.

To investigate whether the TTV DNA load reflected immune system impairment in COPD patients, we evaluated whether the viral load was associated with differences in lung function. Statistical analysis revealed that the TTV DNA load at admission was significantly correlated with the RV/TLC during acute exacerbation (*r* = 0.378, *p* = 0.030) (Figure 2A). Moreover, significant inverse correlations were found between the TTV DNA load at discharge and FEV1/pre and FEV1/FVC in remission (*r* = −0.484, −0.432, *p* = 0.011, 0.024, respectively) (Figures 2B,C, respectively). No significant correlation was observed between the TTV DNA load and other spirometry measures.

### Sputum Load of TTV DNA Is Related to COPD Disease Severity

**Figure 3:**
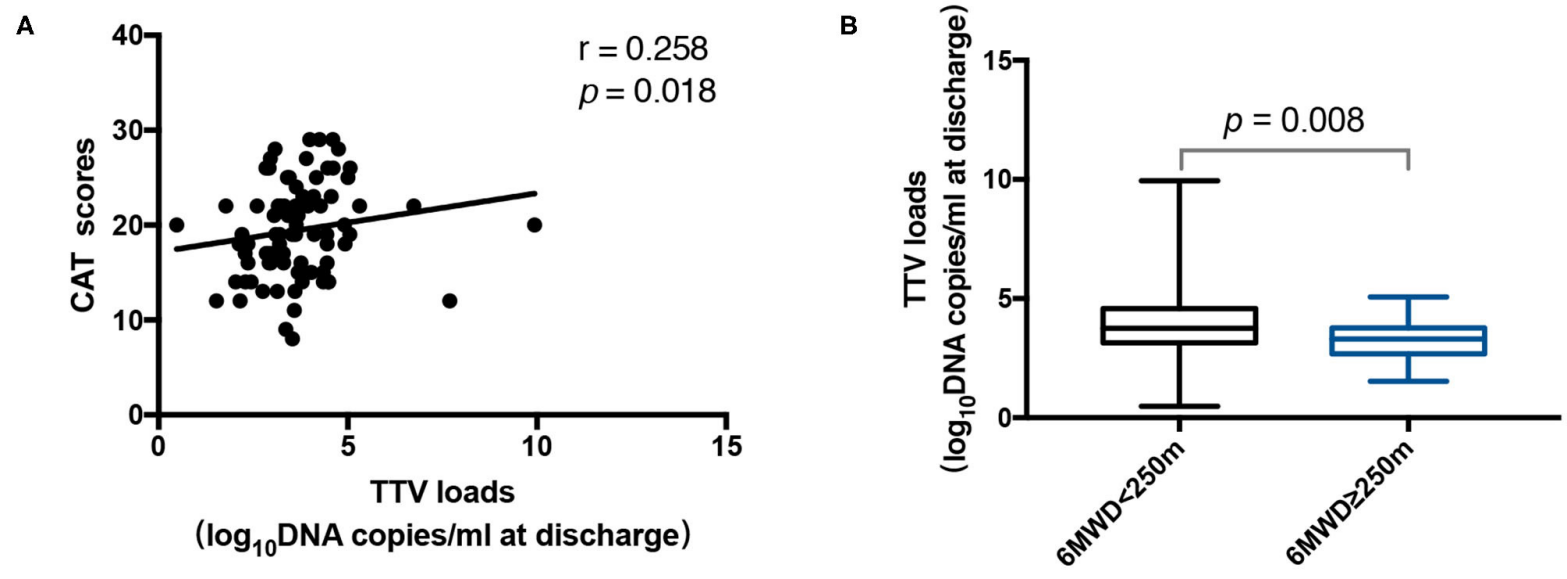
**(A)** Correlation between the Torque Teno virus (TTV) DNA load at discharge and the CAT scores in 84 patients. **(B)** TTV DNA load at discharge for 46 patients with 6 MWD < 250 m and 38 patients with 6 MWD ≥ 250 m. The correlation between the values was assessed by the Spearman's rank test. The Mann-Whitney U test was performed to compare the median values. The *r* and *p*-values are shown. Thick horizontal bars represent the median value of each point.

We also evaluated whether the TTV DNA load was linked with the severity of COPD. There was a neutral connection between the TTV DNA load at discharge and the CAT score (*r* = 0.258, *p* = 0.018) (Figure 3A). Three hundred and fifty meter is usually used as a cutoff value for the 6 MWD (23–25), but considering that most of the patients in our study were below this value, we further used 250 and 150 m as cutoff values to divide the patients into groups and analyzed the differences in TTV DNA load between groups. Finally, it was found that the TTV DNA load at discharge among patients with 6 MWD < 250 m was significantly higher (*p* = 0.008) than that among patients with 6 MWD ≥ 250 m (Figure 3B), while no difference was found in TTV DNA load at admission and discharge when 150 m was used as the cutoff value. However, no statistically significant connection was found between the TTV DNA load and the mMRC scores (data not shown).

### TTV DNA Load Is Related to Other Factors

**Figure 4:**
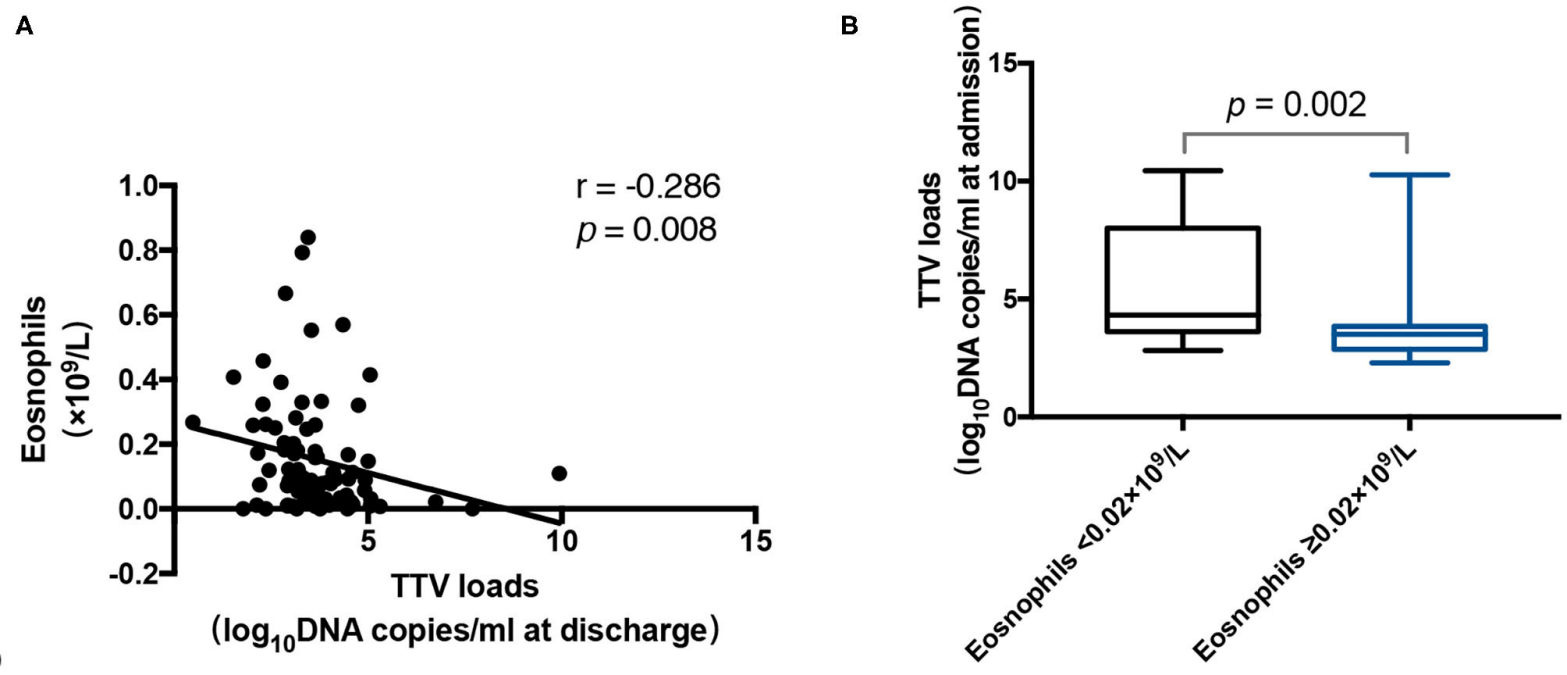
**(A)** Correlation between the Torque Teno virus (TTV) DNA load at discharge and the number of eosinophils in 85 patients. **(B)** TTV DNA load at admission for 14 patients with a number of eosinophils < 0.02 × 10^9^/L and 60 patients with a number of eosinophils ≥ 0.02 × 10^9^/L. The correlation between the values was assessed by the Spearman's rank test. The Mann-Whitney U test was performed to compare the median values. The *r* and *p*-values are shown. Thick horizontal bars represent the median value of each point.

As shown in Figure 4A, the TTV DNA load at discharge was neutrally inversely correlated with the number of eosinophils (*r* = −0.286, *p* = 0.008). Furthermore, the TTV DNA load at admission in patients with eosinophils counts < 0.02 × 10^9^/L was significantly higher (*p* = 0.002) than that in patients with eosinophils counts ≥ 0.02 × 10^9^/L (Figure 4B).

### Prevalence of TTV Genogroups in COPD Patients

TTV was successfully typed in 87 (95.6%) patients. Table 3 shows the frequency of each TTV genogroup and the number of TTV genogroups. TTV genogroups 1 and 4 were the most common, followed by genogroups 3 and 5, and genogroup 2 was rather rare. Most TTV DNA-positive patients carried four distinct genogroups, and decreasing numbers of patients carried three, five, and two genogroups. No patient harbored only a single TTV genogroup.

**Table 3:** Identity and number of TTV genogroups in patients (*n* = 91).

**Category**	**No. (%) of patients**
**TTV genogroup**	
Genogroup 1	86 (94.5%)
Genogroup 2	13 (14.3%)
Genogroup 3	63 (69.2%)
Genogroup 4	86 (94.5%)
Genogroup 5	61 (67.0%)
**No. of TTV genogroups**	
2	9 (9.9%)
3	31 (34.1%)
4	37 (40.7%)
5	10 (11.0%)

*TTV, Torque Teno virus*.

We found ten combinations after analyzing all possible detection. By measuring viral loads at admission in each group, it appeared that the highest TTV DNA load was found in the group containing G1 + G3 + G4 + G5 (3.8 log_10_ copies/ml, IQR 3.5–4.4 log_10_ copies/ml), followed by the two groups containing G1 + G2 + G3 + G4 + G5 (3.7 log_10_ copies/ml, IQR 3.3–4.2 log_10_ copies/ml) and G1 + G4 + G5 (3.4 log_10_ copies/ml, IQR 2.9–4.3 log_10_ copies/ml). Furthermore, the TTV DNA load at admission in the group containing G1 + G3 + G4 + G5 was significantly higher (*p* = 0.042) than that in the group containing G1 + G3 + G4.

Furthermore, we evaluated whether the number of TTV genogroups was associated with the viral load at admission. The median TTV DNA loads at admission in the patients with two, three, four, and five genogroups were 3.0, 3.0, 3.8, and 3.8 log10 copies/ml, respectively. Furthermore, we compared the TTV DNA load in patients with different number of genogroups, and found that the TTV DNA load in patients carrying four genogroups was significantly higher (*p* = 0.017) than that in patients carrying three genogroups.

### Relationship Between TTV Genogroups and Lung Function, COPD Assessment Scores, and Others

We then separated the patients into four groups based on the number of TTV genogroups they harbored. Overall, there was a critically significant difference between the groups for drinking status (*p* = 0.007). Furthermore, it appeared that more TTV genogroups were detected in patients with diabetes mellitus and metabolic comorbidity (*p* = 0.023, 0.001, respectively). No statistically significant difference was noticed between groups for age, FEV1/FVC, FEV1/pre, GOLD grade, mMRC, 6 MWD or CAT score (Table 4).

**Table 4:** TTV genogroups and clinical characteristics of patients (*n* = 87).

	**No. of TTV genogroups**				
**Characteristics**	**Two (*n* = 9)**	**Three (*n* = 31)**	**Four (*n* = 37)**	**Five (*n* = 10)**	***p-*value**
Age^a^	71.0 ± 8.1	70.6 ± 9.6	72.2 ± 8.4	72.6 ± 7.6	0.859
Pack-years	15 (0–30)	30 (0–40)	30 (21–50)	30 (28–50)	0.150
Current smoker	2 (22.2%)	10 (32.3%)	14 (37.8%)	5 (50.0%)	0.607
Drinking status					0.007
No	4 (44.4%)	26 (83.9%)	32 (86.5%)	5 (50.0%)	
Once	1 (11.1%)	2 (6.5%)	2 (5.4%)	0 (0.0%)	
Current	4 (44.4%)	3 (9.7%)	3 (8.1%)	5 (50.0%)	
**Comorbidities**					
Diabetes mellitus	1 (11.1%)	2 (6.5%)	4 (10.8%)	5 (50.0%)	0.023
Metabolic comorbidity	1 (11.1%)	3 (9.7%)	4 (10.8%)	6 (60.0%)	0.001
Hyperlipidemia	0 (0.0%)	1 (3.2%)	0 (0.0%)	2 (20.0%)	0.064
Coronary heart disease	3 (33.3%)	9 (29.0%)	9 (24.3%)	2 (20.0%)	0.891
Chronic kidney disease	0 (0.0%)	1 (3.2%)	1 (2.7%)	0 (0.0%)	1.000
FEV1/FVC, %	40.80 (38.35–62.00) (*n* = 9)	49.98 (40.75–59.83) (*n* = 28)	47.87 (39.78–52.91) (*n* = 31)	44.48 (40.46–49.62) (*n* = 10)	0.477
FEV1, % predicted	26.00 (20.05–45.15) (*n* = 9)	33.55 (21.88–46.98) (*n* = 28)	31.69 (21.00–54.00) (*n* = 31)	25.90 (17.70–32.78) (*n* = 10)	0.346
GOLD grade^b^					0.442
1	0 (0.0%)	0 (0.0%)	1 (2.7%)	0 (0.0%)	
2	2 (22.2%)	5 (16.1%)	5 (13.5%)	0 (0.0%)	
3	1 (11.1%)	13 (41.9%)	11 (29.7%)	4 (40.0%)	
4	6 (66.7%)	13 (41.9%)	20 (54.1%)	6 (60.0%)	
6 MWD < 250 m	5 (55.6%)	11 (36.7%)	24 (64.9%)	7 (70.0%)	0.093
mMRC > 2	4 (44.4%)	16 (51.6%)	25 (67.6%)	7 (77.8%)	0.273
CAT^a^	18.89 ± 5.35	18.16 ± 5.05	20.78 ± 4.88	19.89 ± 4.60	0.187

*Clinical values represent the mean ± standard deviation (SD) or median values (interquartile range, IQR). Continuous values were compared by using the Kruskal-Wallis test, and Fisher's exact test or the chi-square test were used to assess the proportions between the groups, unless otherwise indicated*.

a *Variables were analyzed by using one-way analysis of variance (ANOVA)*.

b *Variables were analyzed by using The chi-square test for trend*.

*TTV, Torque Teno virus; FEV1, forced expiratory volume in 1 s; FVC, forced vital capacity; 6 MWD, 6-min walk distance; mMRC, modified British medical research council; CAT, COPD assessment test*.

## Discussion

For the first time, we demonstrated the distribution of TTV in the lower respiratory tracts of COPD patients. We found that TTV detection is highly prevalent in COPD patients, in which multiple TTV genotype combination is frequent. Also, the sputum load of TTV could serve as a lung function and disease severity index in COPD patients.

We discovered a high prevalence of TTV in COPD patients (95.6%), which is in keeping with previous findings from studies of respiratory diseases (prevalence of 87% in children who were diagnosed with acute respiratory diseases, 85% in children with persistent or recurrent pneumonia and 93% in children with asthma) (15–17, 26). The presence of TTV is quite common and frequently tends to persist, as the viral load barely declines during follow-up. It has come to light that some molecular markers indicate the occurrence of the replication of TTV in the lungs (27, 28). A research conducted by Maggi et al. revealed that patients with TTV-positive nasal detection did not harbor TTV viremia. However, cases with TTV viremia did not always reveal positive exposure in the nose (15). These findings demonstrated that TTV might continually replicate in the respiratory system and that the respiratory tract might be an entrance and a reservoir of distribution for TTV.

Multiple TTV genotype combination have been reported as frequent events in healthy adults (29, 30). Consistent with our study, multiple TTV genotypes were detected in all TTV DNA-positive patients, and genotype 1 was the most frequently detected; furthermore, most patients carried four distinct genogroups (40.7%), which is more than previously reported (34% carried two distinct genogroups according to plasma samples) (22, 26). As the number of harbored genogroups increased, the viral loads increased. However, the association between virus loads and the number of TTV genogroups was not linear. Genogroup 2 had a relatively low infection rate, and similar results have been reported in another study (22, 31).

Compared to previous researches, our study demonstrates that the detection rate of TTV in COPD is higher than that of other respiratory diseases. We assumed that the impaired immune system in COPD patients might contribute to the high prevalence of TTV. TTV loads may reflect the immunocompetence of hosts (12, 32, 33), as higher TTV DNA loads are correlated with a more intense immunosuppressive state. High TTV loads may indirectly lead to more severe and more frequent infections by reducing cellular and humoral immune responses. Another piece of evidence linking TTV load to the systemic immune status is that TTV load is associated with the CAT score, although the correlation index was relatively low (*r* < 0.3), which may due to the relatively small sample size. The CAT score is a comprehensive evaluation of the systemic state of COPD patients, which to some extent also reflects the systemic immune capacity of COPD patients. Furthermore, we found that compared with TTV detection at admission, the number of patients with detectable TTV at discharge increased and TTV viral load was moderately elevated. During hospitalization, most patients were treated with corticosteroids, which led to a decreased immune response and as a result, increased TTV detection rates and viral load. To assess whether the type of TTV is correlated with pulmonary function and the severity of the disease, we detected the genogroups of TTV in the sputum of all TTV-positive patients. Overall, there was a significant association between the TTV genogroups and drinking status, diabetes mellitus, and metabolic comorbidity. However, there was no significant correlation between the types of TTV genogroups and spirometric indices and the severity of the disease, which is consistent with previous reports (17).

We then evaluated whether sputum TTV loads were associated with pulmonary function. We found that the TTV load during acute exacerbation was significantly correlated with the RV/TLC during acute exacerbation; chronic inflammation leading to structural destruction and small airway occlusion is an accepted pathological basis of COPD (21), resulting in airway closure that may be related to an increase in the RV/TLC ratio. Moreover, significant inverse correlations were observed between the TTV DNA load in the remission stage and FEV1/pre and FEV1/FVC in the remission stage, which indicated the degree of the obstruction of the medium and small airways and were reported to be predictive spirometric markers for delicate airway dysfunction (34, 35). TTV loads were also associated with decreases of most of the spirometric indices, although there was no statistical significance. TTV may disrupt the systemic or local immune system, leading directly to subtle airway alterations. Due to the short of an experimental environment to support TTV growth, the physiological characteristics of TTV are still unclear. Further research is required in this area.

In the study, we demonstrated the relationships between TTV and pulmonary function and the disease severity of COPD. Previous studies on TTV have mainly focused on immunosuppression in patients after transplantation. The immune status of COPD patients is impaired, so our study also confirms the relationship between TTV and the immune system from another perspective. Our study also has some limitations. First, TTV DNA was not determined in bronchoalveolar lavage fluid and plasma, and the distribution of TTV virus in specimens from different sources could not be compared. Furthermore, the study suggested that the TTV DNA load at discharge was neutrally inversely correlated with the number of eosinophils. We also found that there was an inverse correlation between the number of eosinophils and the severity of disease (data not shown). However, further research is expected to illustrate the association between TTV infection, the number of eosinophils and the pathogenesis of COPD. Finally, our study was limited in that the TTV viral load was not measured in healthy people, which may provide us with basic information on the distribution of TTV virus. And, researches included larger population are warranted.

## Conclusion

In the current study, the TTV viral load is positively associated with lung function and disease severity in COPD patients. Based on the high prevalence and the steady-state distribution of TTV, we believe this underlines the possible utility of sputum TTV DNA loads as a novel biomarker for the clinical management of COPD.

## Data Availability Statement

The raw data supporting the conclusions of this article will be made available by the authors, without undue reservation.

## Ethics Statement

The studies involving human participants were reviewed and approved by the Ethics Committee of Peking University People's Hospital. The patients/participants provided their written informed consent to participate in this study.

## Author Contributions

YXi, QX, YZ, and ZG: conception and design. YXi, WJ, JW, YY, LZ, and QX: acquisition, analysis, or interpretation of data. YXi and QX: drafting the manuscript for important intellectual content. YXi, YXu, and YY: statistical analysis. WJ, JW, XD, GF, and QX: administrative, technical, or material support. YZ and ZG: study supervision. All authors contributed to the article and approved the submitted version.

## Conflict of Interest

The authors declare that the research was conducted in the absence of any commercial or financial relationships that could be construed as a potential conflict of interest.
